# Integrating metabolomics and transcriptomics to investigate the effects of changes in renal adenosine 5′-monophosphate and arginine metabolism on the mTOR pathway under chronic hypoxia

**DOI:** 10.1515/med-2026-1436

**Published:** 2026-06-15

**Authors:** Hong Liang, Kang Song

**Affiliations:** Department of Basic Medical Sciences, Medical College, Qinghai University, Xining, China; Endocrinology Department, Qinghai Provincial People’s Hospital, Xining, Qinghai, China

**Keywords:** adenosine 5′-monophosphate, arginine, hypoxia, renal, mTOR

## Abstract

**Objectives:**

Hypoxia leads to pathophysiological changes in the body. Kidneys are important organs essential for maintaining normal body function. The metabolic changes in renal under chronic hypoxia are still unclear. To investigate how chronic hypobaric hypoxia alters renal metabolites and mTOR-related signalling pathways in mouse kidney.

**Methods:**

The tests used renal tissues from mice maintained for 4 weeks in hypoxia and normoxia at altitudes of 5000 m and 50 m. Mice renal at different altitudes were screened for differential metabolites and genes using non-targeted metabolomics and transcriptomics. The effect of hypoxia on renal tissue metabolic pathways was clarified using Gene Ontology (GO) and Kyoto Encyclopaedia of Genes and Genomes (KEGG) enrichment analyses.

**Results:**

A total of 120 and 79 markedly altered metabolites were identified as differential metabolites in the positive and negative ion modes, respectively. A total of 4155 significantly altered genes were identified, of which 2342 were upregulated and 1813 downregulated in the high-altitude group.

**Conclusions:**

A combined analysis of metabolomics and transcriptomics revealed that adenosine 5′-monophosphate(AMP) and arginine were associated with changes in the renal mTOR signalling pathway under conditions of chronic hypoxia. Our findings on AMP and arginine metabolism and differentially expressed genes(DEGs) provide insights into chronic hypoxia-induced mTOR changes in kidney.

## Introduction

Hypoxia is a typical characteristic of plateau environments. High altitude can be used as a model to simulate hypoxia conditions and study its effects on the human body, but it is also an extreme environment that directly affects millions of people who travel to high altitudes or live in high-altitude locations for prolonged periods [1].

The kidney is one of the most complex excretory organs that can regulate the excretion of water, salts, and waste through a highly dynamic range of urine volumes, thereby protecting the internal environment [2]. Chronic intermittent hypoxia exposure induces kidney injury in growing rats [3] and histological kidney damage is associated with increased growth factor expression during obstructive sleep apnoea tests in mouse models [4]. Furthermore, hypoxia severely affects cellular metabolism and several associated physiological responses [5]. Hypoxia signalling is predominately associated with the mechanistic target of the rapamycin complex (mTOR), the main cellular energy- and nutrient-sensing pathway.

The serine/threonine kinase mTOR controls cell growth, proliferation, and survival in response to nutrition, growth factors, and stress [6]. Beyond these core cellular functions, mTOR serves as regulator of renal cell homeostasis and autophagy, which are critical for maintaining normal renal physiological activities [6], [7], [8]. Moreover, mTOR is closely implicated in the progression of various renal disorders, including renal injury, glomerular disease, polycystic kidney disease, and kidney transplant rejection, acting as a key regulatory node in the pathogenesis of these conditions [7]. In most mammals and humans, arginine is endogenously synthesised via the intestinal-renal axis, which constitutes a vital metabolic pathway for amino acid supply in renal tissues [9]. Arginine participates in diverse cellular physiological processes, ranging from protein synthesis to immune regulation, and has been identified as a direct activator of mTOR signalling [10]. Given the close interplay between arginine metabolism and mTOR pathway, it remains poorly understood how chronic hypoxia-induced alterations in renal AMP and arginine levels modulate the downstream mTOR signalling cascade.

Transcriptomics enables comprehensive profiling of transcriptional sequences in tissues or organs, allowing real-time identification of differential gene expression patterns and prediction of disease-related molecular mechanisms and signalling pathways [11]. Complementarily, liquid chromatography-mass spectrometry (LC-MS) is a powerful analytical tool for qualitative and quantitative detection of tissue metabolites, facilitating reliable comparative analysis of metabolic profiles between different experimental groups [12]. Despite the unique advantages of LC-MS in metabolomic characterisation, this technique alone cannot capture dynamic changes in gene expression levels. This inherent limitation restricts its ability to fully elucidate the hypoxia-specific biological characteristics associated with the renal mTOR signalling pathway, highlighting the necessity of integrating multi-omics strategies in our study.

Therefore, metabolite and gene expression profile changes can be investigated and interpreted by combining metabolomic and transcriptomic approaches. With the advancement in systems biology, multi-omics integration, including the metabolome and transcriptome, has recently been used to investigate hypoxia-related mechanisms. Na et al. identified the molecular targets of copper in pig kidneys using metabolomics and transcriptomics [13].

In the current study, we assessed the effects of hypoxia on kidney tissue using transcriptomic and metabolomic analyses. An untargeted metabolomic analysis was conducted on renal tissue to detect metabolic changes, followed by a transcriptomic analysis to identify differentially expressed genes(DEGs) under hypoxic conditions. Network and pathway analyses were used to clarify the relationship between adenosine 5′-monophosphate(AMP) and arginine and DEGs after hypoxia treatments and to investigate hypoxia-related metabolic characteristics affecting the mTOR signalling pathway in renal tissue using metabolomic and transcriptomic approaches.

## Materials and methods

### Animals

In this study, 16 male C57BL/6J mice aged six weeks were acquired from SPF Biotechnology Co., Ltd., (Beijing, China). After one week of adaptive feeding, the animals were randomly divided into two groups. Mice in the low-altitude kidney group (LK) were housed at an altitude of 50 m, whereas those in the high-altitude kidney group were exposed to conditions simulating an altitude of 5,000 m in a hypobaric chamber (Guizhou Feng Lei Oxygen Chamber Co., Ltd., Guizhou, China). The two groups were maintained for 4 weeks at a temperature of 22 ± 2 °C, humidity of 45–55 %, and a 12-h light/dark cycle.

### Transcriptomic analysis

#### RNA extraction

Total RNA from the kidney tissue was extracted using a TRIZOL reagent kit. Six independent biological replicates (n=6) were used per experimental group, with each replicate representing a single mouse kidney. After RNA extraction, the A260/A280 absorbance ratio of RNA samples was determined using a Nanodrop ND-2000 spectrophotometer, and the RNA integrity number (RIN) was measured with an Agilent Bioanalyzer 4,150. The RIN value of each sample is provided in the supplementary materials (Supplementary Table 1).

#### Library construction and sequencing

Paired-end (PE) libraries were prepared according to the manufacturer’s instructions of the ABclonal mRNA Seq Lib Prep Kit (ABclonal, China). The mRNA was extracted from 1 μg of total RNA using oligo (dT) magnetic beads and then fragmented in ABclonal First Strand Synthesis Reaction Buffer. The first strand of cDNA was then synthesised using random primers and reverse transcriptase (RNase H) with mRNA fragments as templates, followed by the second strand of cDNA using DNA polymerase I, RNAseH, buffer, and dNTPs. The synthesised double-stranded cDNA fragment was ligated to a splice sequence for amplification by polymerase chain reaction.

The original image data file obtained by high-throughput sequencing was analysed by CASAVA Base Calling, converted into the original sequencing sequence, and stored in FASTQ file format, which contains the corresponding sequence and quality information. The original sequence contained reads with low-quality joints. To ensure the quality of subsequent analysis, the joint sequence was removed, and the number of reads with low-quality (the average base mass value is less than 20) and N (N represents the undetermined base) larger than 5 were filtered out, and clean reads suitable for subsequent analyses were obtained. The data quality of each sample is shown in Supplementary Table 2. Mapped reads were analysed to compare the reference genome and clean reads using the HISAT2 software (http://daehwankimlab.github.io/hisat2/). Reads vs. reference genome is shown in Supplementary Table 3. The Recount FPKM value for each factor was calculated for each sample using the feature Counts software. DESeq2 (http://bioconductor.org/packages/release/bioc/html/DESeq2.html) was used for assessing differential gene expression in the two groups. The default threshold values for screening DEGs were: |log_2_FC| >1 and P adj<0.05. Finally, DEGs were used for GO and KEGG pathway enrichment analyses.

### Extraction of metabolites and metabolomic analysis

#### Sample extraction

Metabolite extraction and metabolomics analyses were performed by Shanghai Applied Protein Technology Biotechnology Co., Ltd. Each group included 6 independent biological replicates, corresponding to 6 individual mouse kidneys per group. The two mouse groups were transferred to the same location for tissue sample collection. Samples were slowly ground at 4 °C, and proper amounts of each sample were mixed, ultrasonicated on ice for 30 min, kept at a low temperature for a while, and then centrifuged at 14,000×*g* at 4 °C for 20 min. Finally, the supernatant was collected and vacuum-dried for later use. The vacuum-dried samples were diluted in 100 μL acetonitrile, mixed by continuous vortexing, and centrifuged at high speed for 15 min at a low temperature. The supernatant was collected for mass spectrometry detection and analysis.

#### Ultra-high performance liquid chromatography (UHPLC) system-QTOF/MS analysis

The Agilent 1290 Infinity LC UHPLC HILIC column was used for sample separation. The column temperature was 25 °C; the flow rate was 0.5 mL/min; the injection volume was 2 μL; the mobile phase consisted of water + 25 mM ammonium acetate + 25 mM ammonia water (A) and acetonitrile (B). The gradient elution procedure was 0 to 0.5 min, 95 % B. Then, a linear gradient of 95 to 65 % B from 0.5 to 7 min was applied, followed by 65 to 40 % B from 7 to 8 min, B was maintained at 40 % from 8 to 9 min, and then a linear gradient of 40 to 95 % B was applied from 9 to 9.1 min. Finally, mobile phase B was maintained at 95 %. from 9.1 to 12 min. The samples were placed in an autosampler at 4 °C throughout the analysis. The samples were consecutively analysed randomly to minimise the influence of the instrument detection signal fluctuation.

The samples were separated by UHPLC coupled with a Triple TOF 6600 mass spectrometer (AB SCIEX), and detection was carried out by electrospray ionisation (ESI) in positive and negative ion modes.

Raw data were converted into mzXML using ProteoWizard, and then peak alignment, retention time correction, and peak area extraction were performed using the XCMS software. Metabolite structures were identified through the analysis of preprocessed data extracted by XCMS, along with the evaluation of experimental data quality and final data. Overall data analysis included univariate and multivariate statistical analysis, screening for different metabolites, correlation analysis of different metabolites, and KEGG pathway analysis. Student’s t-test was used to calculate the original p-values of metabolites. The Benjamini-Hochberg (BH) approach was applied to adjust raw p-values for false discovery rate (FDR) correction. Differential metabolites were strictly screened with the following thresholds: VIP>1, raw p<0.05, and adjusted P (FDR) <0.05.

#### Integrative analysis of transcriptomics and metabolomics

Spearman correlation coefficient analysis was used to evaluate correlations between significantly different genes and metabolites in the samples. The R (version 3.6.3) language and Cytoscape (version 3.8.2) software were used for matrix heat mapping, hierarchical clustering, and correlation network analysis to explore the interaction between genes and metabolites from multiple perspectives.

### Statistical analysis

p<0.05 and |log_2_ FC| >1 were set up as criteria for DEGs. OPLS-DA VIP >1 and Padj<0.05 were set up as criteria for differential metabolites. Data was analysed using GraphPad Prism 8.0 (GraphPad Software, San Diego, CA, USA). Independent sample *t*-test was used for counting data. p<0.05 was considered statistically significant.

**Ethics statement:** The authors confirm that the ethical policies of the journal, as noted on the journal’s author guidelines page, have been adhered to. All mouse treatments and experimental protocols have been approved by the Institutional Animal Care and Use Committee of Qinghai University in compliance with the Animal Management Rules of the Chinese Ministry of Health (NO. 2022–97).

**Informed consent:** Not applicable.

## Results

### Identification of DEGs in kidney

**Figure 1:**
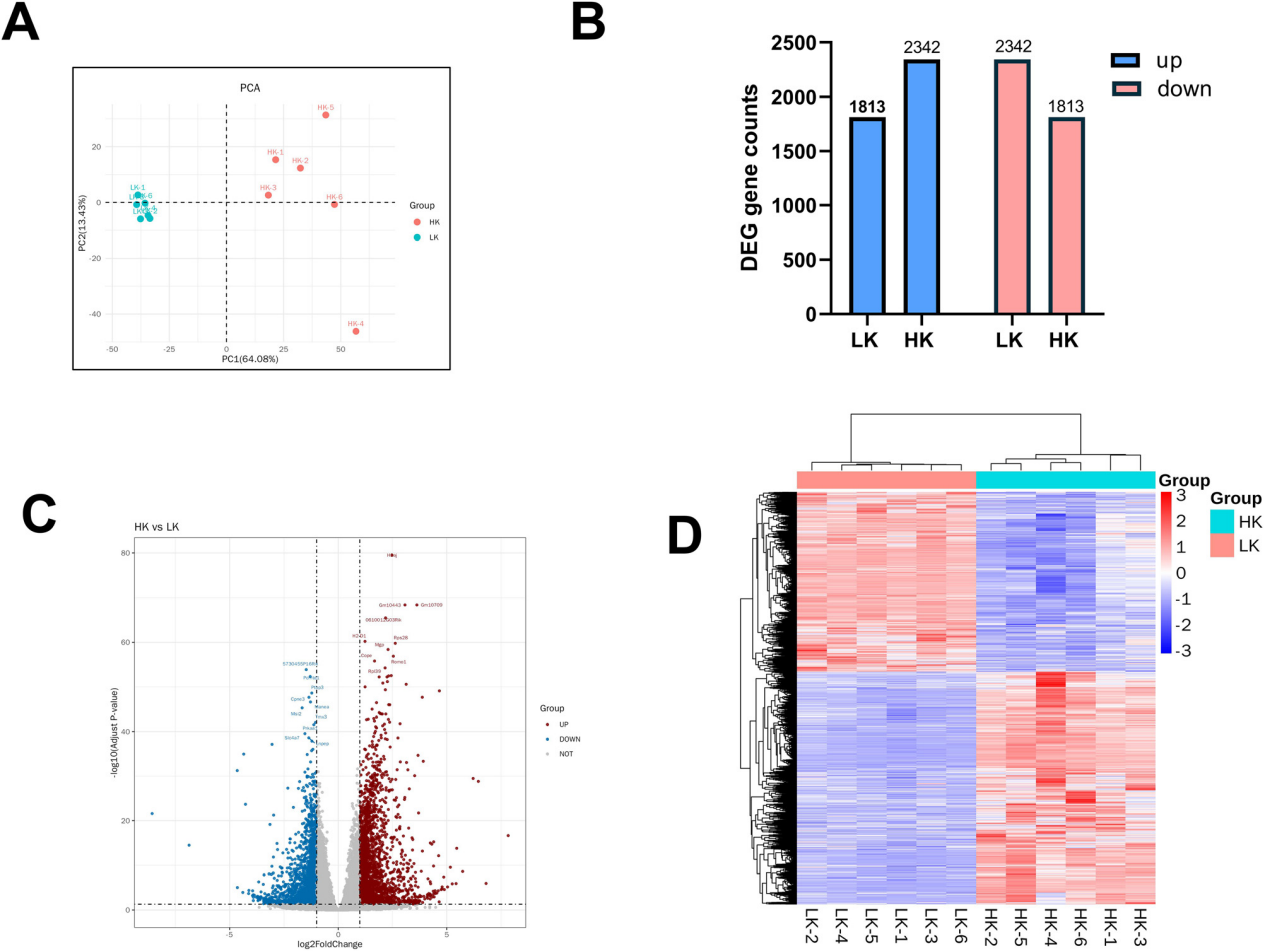
Effects of hypoxia on renal transcriptomics. (A) Two-dimensional PCA plot (B) DEGs between the two groups. (C) Volcano plot of differential gene expression distribution. The abscissa represents the fold change of gene expression in different experimental groups or different samples. The ordinate indicates the statistical significance of the changes in gene expression. The scatter points in the figure represent individual genes, grey dots indicate genes with no significant difference, red dots indicate upregulated genes with significant difference, and blue dots indicate downregulated genes with significant difference. (D) Two groups of differential gene cluster map. LK: 50 m normoxia for 4 weeks. HK: simulated 5,000 m hypoxia for 4 weeks.

To identify kidney-related DEGs, transcriptomic profiles of mouse kidneys under normal or hypoxic conditions were analysed after RNA sequencing. Principal components analysis (PCA) showed that the renal gene expression profile of normoxic mice was significantly different from that of hypoxic mice. PC1 was 64.08 %, PC2 was 13.43 %, and PC3 was 5.33 %, as shown in Figure 1A. The structural sequence, low-quality reads and unascertained reads with more than five bases were removed. A total of 32,906 genes were identified, including 4,155 differential genes, among which the HK group had 2,342 upregulated genes and 1,813 downregulated genes and LK group had 1,813 upregulated genes and 2,342 downregulated genes (Figure 1B). The volcano plot was used to visualise the distribution and expression changes of the differential genes in the two group samples (Figure 1C). Similar results were also detected in the cluster analysis heatmap (Figure 1D).

### GO and KEGG enrichment analyses

**Figure 2:**
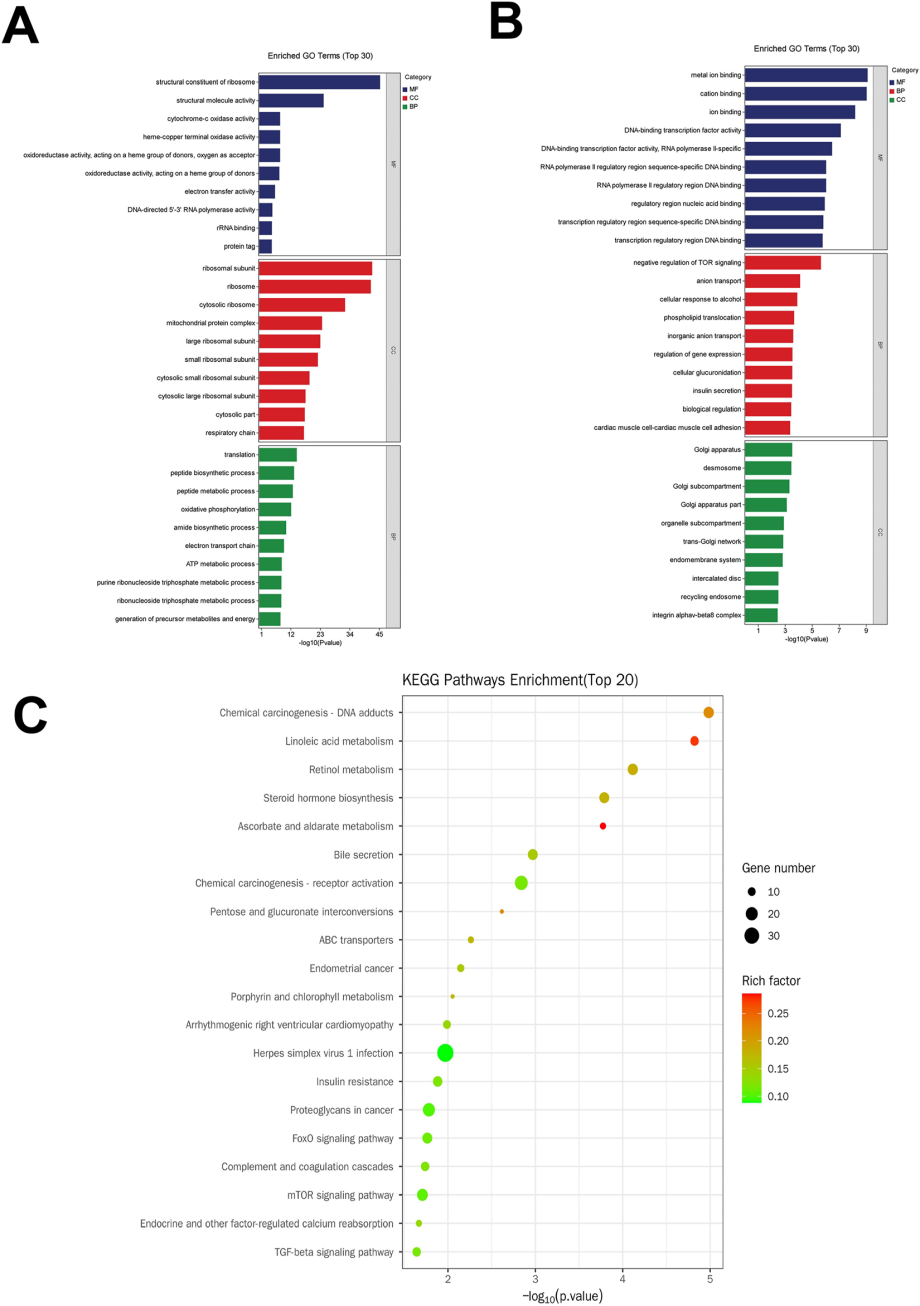
Significantly affected pathways in renal tissue under hypoxia. (A) List of GO terms with the top 30 upregulated and (B) downregulated DEGs. (C) The top 20 downregulated pathways for KEGG enrichment.

GO functional characterisation includes biological process (BP), cell component (CC), and molecular function (MF). The top 30 up- and downregulated DEGs of HK vs. LK are shown in Figure 2A and B, respectively. Our results showed that negative regulation of TOR signalling was the most prominent GO BP term among the top 10 downregulated processes (Figure 2B). To identify the enriched pathways of these DEGs, KEGG pathway analysis was performed. Figure 2C showed the top 20 downregulated pathways for KEGG enrichment, among which the mTOR pathway was one of the most significantly enriched pathways among the downregulated pathways (Figure 2C). Among the 33 DEGs in mTOR signaling pathway, 16 were downregulated and 17 were upregulated (Table 1).

**Table 1:** 33 DEGs in the mTOR pathway.

Gene_ID	Gene	log2FC	p-Value	*P*-adj	Trend
ENSMUSG00000002413	*braf*	−1.0079	1.1869e-11	1.5761e-10	Down
ENSMUSG00000003068	*stk11*	1.0501	2.7808e-24	1.9054e-22	Up
ENSMUSG00000004285	*Atp6v1f*	1.3143	2.3754e-26	2.1718e-24	Up
ENSMUSG00000010057	*Nprl2*	1.0043	9.7768e-15	1.9548e-13	Up
ENSMUSG00000010797	*Wnt2*	1.6471	0.00013554	0.00059519	Up
ENSMUSG00000013663	*Pten*	−1.0938	6.3954e-12	8.8546e-11	Down
ENSMUSG00000015957	*Wnt11*	1.0188	0.0012946	0.004687	Up
ENSMUSG00000019970	*Sgk1*	1.0273	9.3032e-07	5.8443e-06	Up
ENSMUSG00000020108	*Ddit4*	2.517	2.6106e-16	6.4065e-15	Up
ENSMUSG00000022297	*Fzd6*	−1.2406	4.5672e-25	3.5216e-23	Down
ENSMUSG00000022419	*Deptor*	−1.5775	6.9096e-22	3.5426e-20	Down
ENSMUSG00000022812	*Gsk3b*	−1.1462	4.9285e-30	6.7073e-28	Down
ENSMUSG00000024122	*Pdpk1*	−1.1717	1.8501e-08	1.5152e-07	Down
ENSMUSG00000024401	*Tnf*	−2.5923	3.3509e-11	4.1199e-10	Down
ENSMUSG00000024830	*Rps6kb2*	1.0014	0.000109	0.00048693	Up
ENSMUSG00000025499	*Hras*	1.3239	1.1425e-23	7.1788e-22	Up
ENSMUSG00000025665	*Rps6ka6*	−1.5557	3.9572e-14	7.3964e-13	Down
ENSMUSG00000026167	*Wnt10a*	1.9321	0.00018064	0.0007734	Up
ENSMUSG00000028062	*Lamtor2*	1.1446	3.177e-09	2.9384e-08	Up
ENSMUSG00000028278	*Rragd*	−1.0312	8.1093e-10	8.1811e-09	Down
ENSMUSG00000028518	*Prkaa2*	−1.4365	3.6884e-16	8.8558e-15	Down
ENSMUSG00000029071	*Dvl1*	1.3867	8.6283e-24	5.588e-22	Up
ENSMUSG00000031309	*Rps6ka3*	−1.0913	2.3106e-12	3.4359e-11	Down
ENSMUSG00000031490	*Eif4ebp1*	1.7529	9.9459e-24	6.3714e-22	Up
ENSMUSG00000033227	*Wnt6*	2.1291	0.010669	0.031164	Up
ENSMUSG00000034801	*Sos2*	−1.0526	1.3816e-17	4.0356e-16	Down
ENSMUSG00000035027	*Map2k2*	1.4573	1.9472e-29	2.4162e-27	Up
ENSMUSG00000035992	*Fnip1*	−1.4385	8.5075e-33	1.675e-30	Down
ENSMUSG00000036106	*Prr5*	1.5992	5.456e-11	6.4838e-10	Up
ENSMUSG00000047496	*Rnf152*	−1.5324	2.2368e-18	7.2991e-17	Down
ENSMUSG00000050552	*Lamtor4*	2.1965	1.51e-47	1.2484e-44	Up
ENSMUSG00000050697	*Prkaa1*	−1.1282	4.4657e-45	2.806e-42	Down
ENSMUSG00000055980	*Irs1*	−1.6571	1.3075e-08	1.0982e-07	Down

### Changes in the metabolic profile of renal tissue under chronic hypoxia

**Figure 3:**
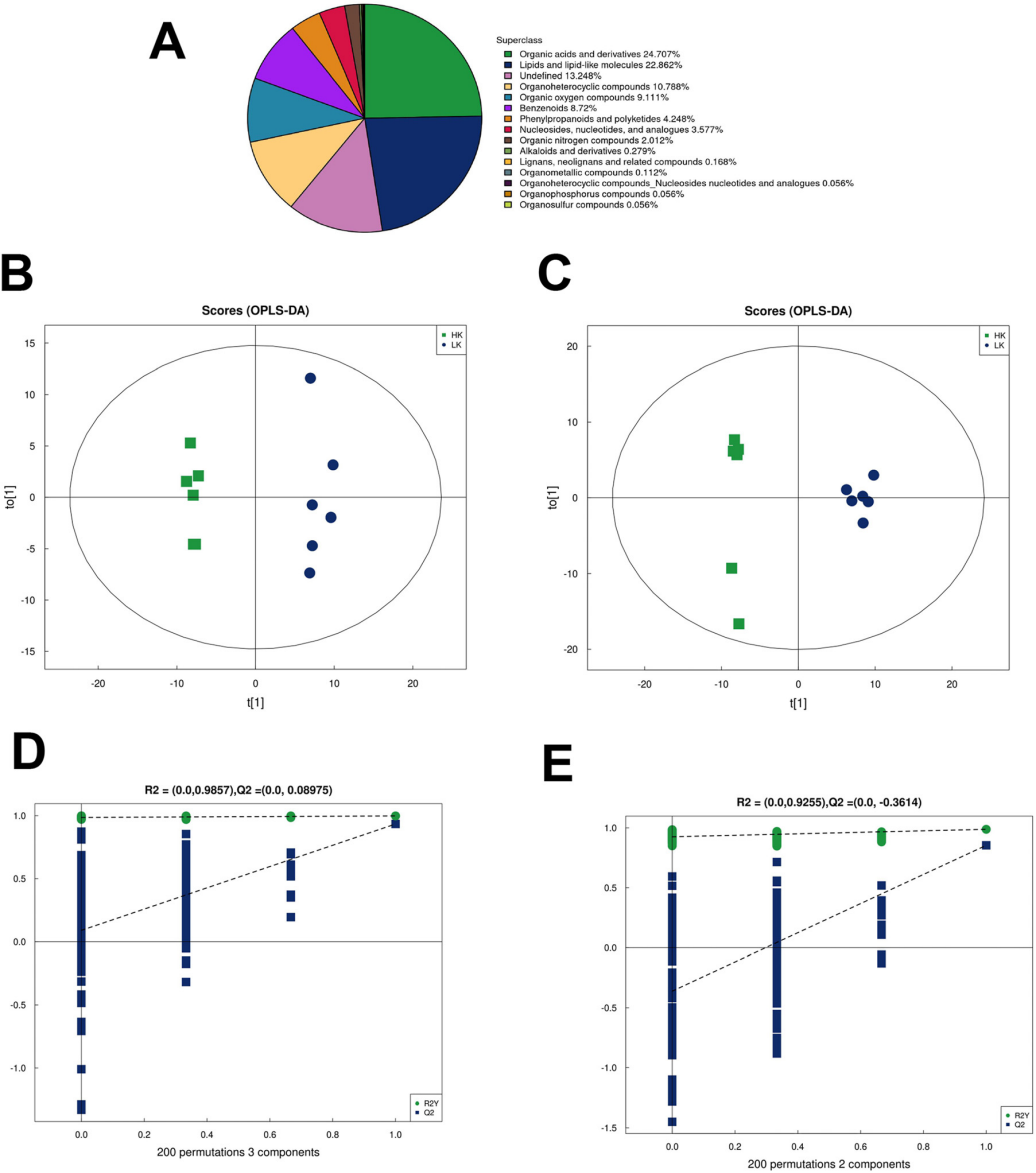
Effects of hypoxia on renal metabolomics. (A) The number and proportion of metabolites identified in each chemical classification. (B) OPLS-DA score plot in positive ion mode. (C) OPLS-DA score plot in negative ion mode. (D) PLS-DA replacement test for positive ion mode, (E) PLS-DA replacement test for negative ion mode. LK:50 m normoxia for 4 weeks. HK: simulated 5,000 m hypoxia for 4 weeks.

**Figure 4:**
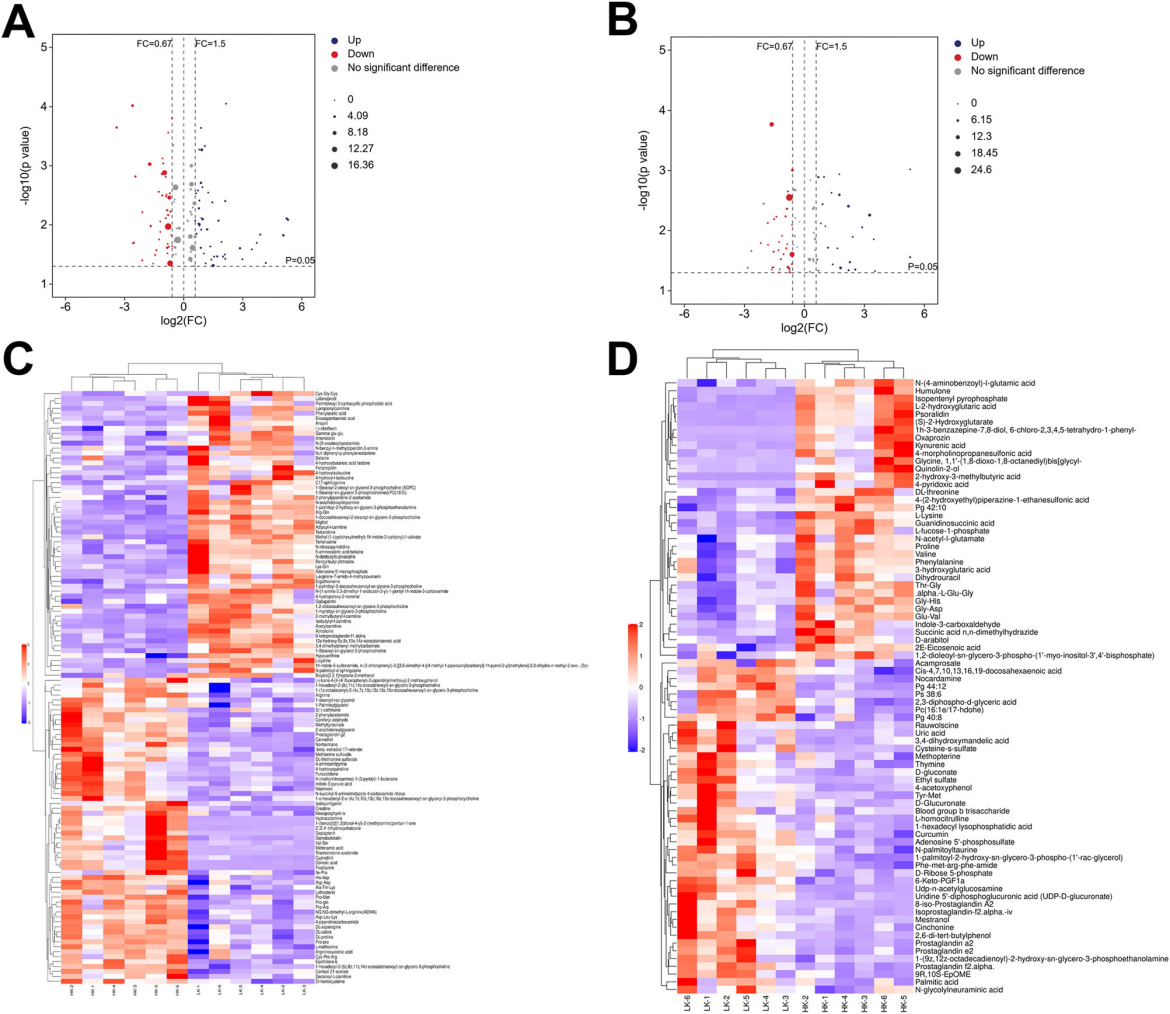
Univariate analysis of renal tissue transcriptomics under hypoxia. (A) Volcano plot in positive ion mode, (B) Volcano plot in negative ion mode. (C) Positive ion pattern significant difference metabolite level clustering heat map. (D) Negative ion pattern significant difference metabolite level clustering heat map. LK: 50 m normoxia for 4 weeks. HK: simulated 5,000 m hypoxia for 4 weeks.

**Figure 5:**
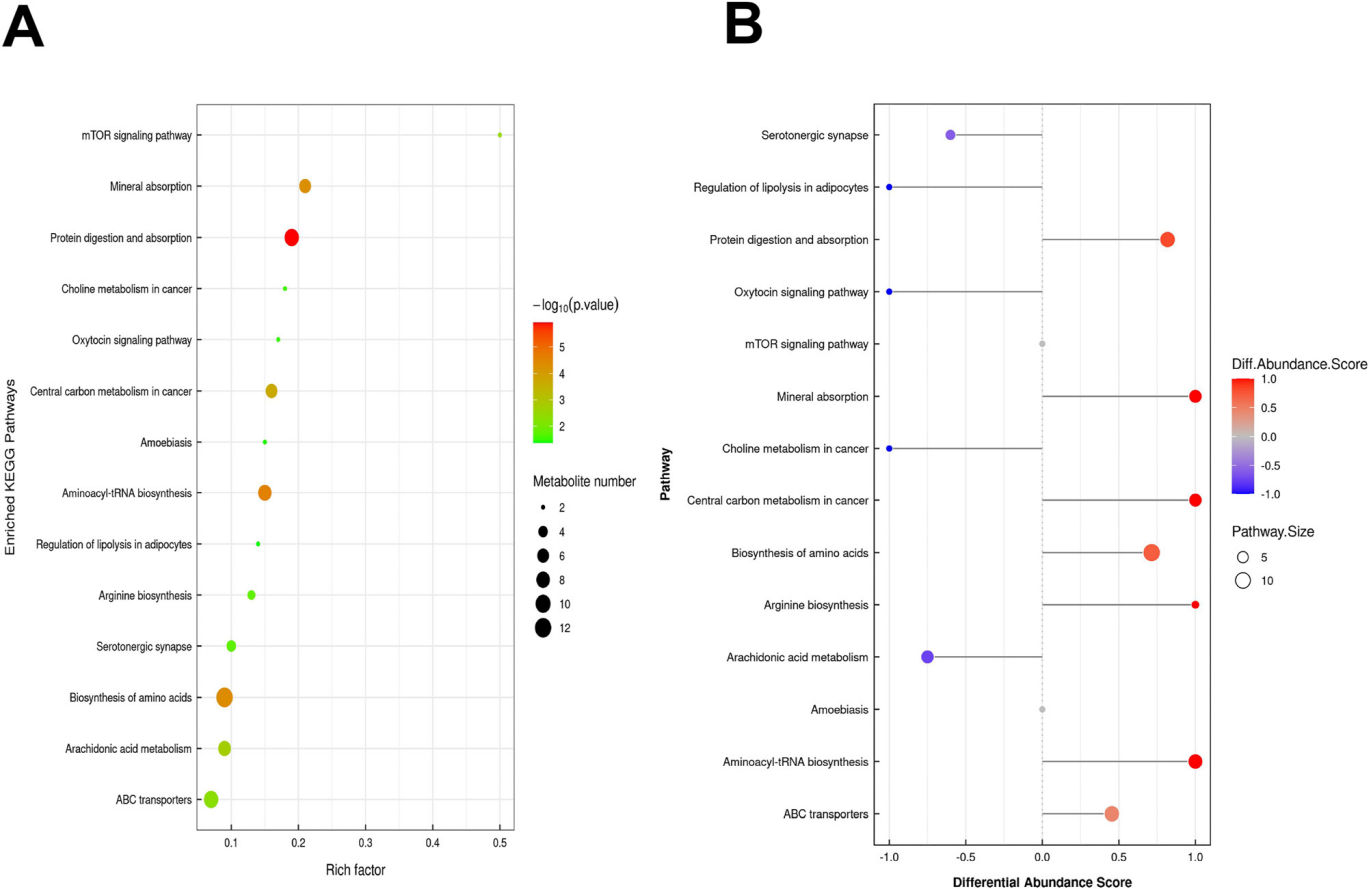
Effects of hypoxia on metabolic pathways in renal tissues. (A) KEGG pathway enrichment pathway map. (B) Differential abundance score plots for all enriched metabolic pathways.

**Figure 6:**
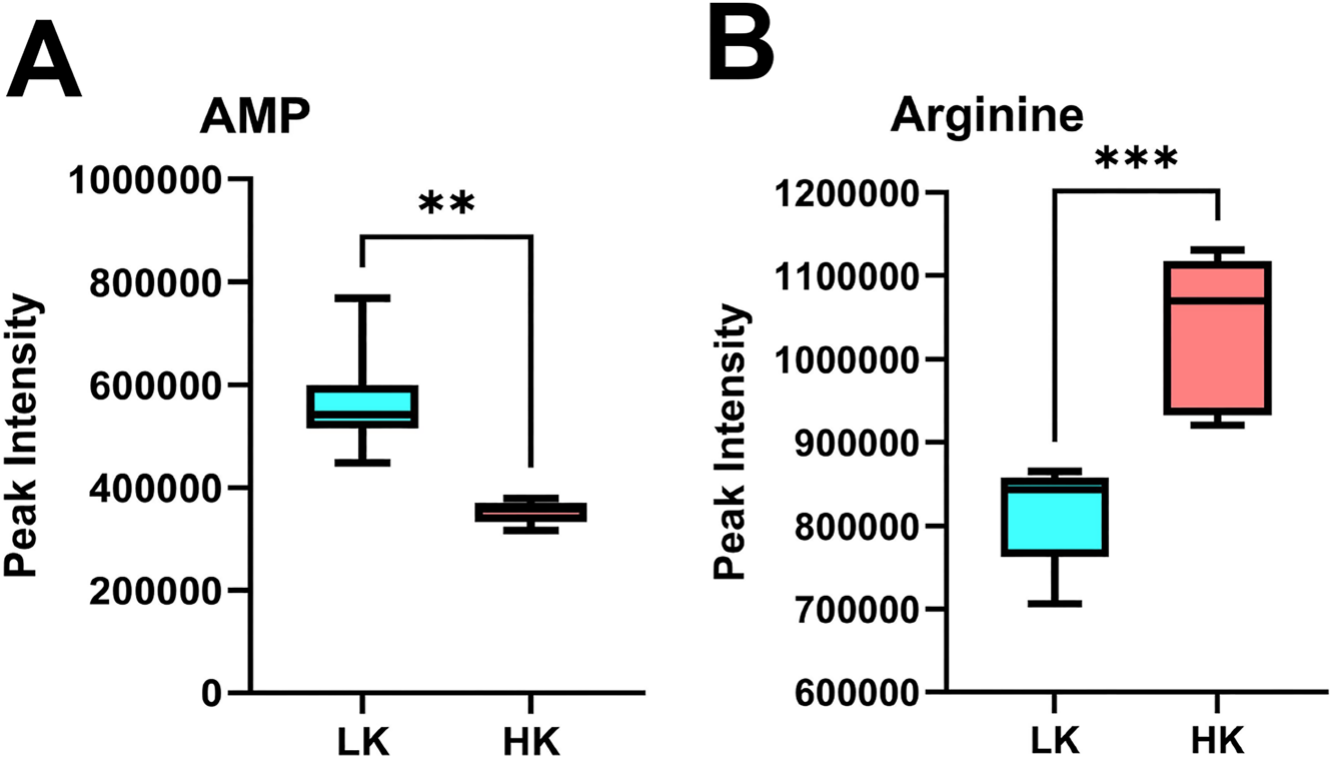
Effects of hypoxia on renal tissues AMP and arginine metabolites. (A) Peak intensity of AMP and (B) arginine in renal tissue. LK: 50 m normoxia for 4 weeks. HK: simulated 5,000 m hypoxia for 4 weeks. n=6 per group, **p<0.01, ***p<0.001.

Six quality control indicators were adopted for evaluation in this experiment (Supplmentary Figure 1–6). The results demonstrated favorable stability of the instrumental analytical system, as well as high stability and reliability of the experimental data. The differences in metabolic profiles obtained in the experiment could reflect the inherent biological differences among samples.

All metabolites identified in this study (metabolites identified combined with positive and negative ions) were classified and counted according to their chemical classification and attribution information. The proportion of various metabolites is shown in Figure 3A, among which organic acids and derivatives accounted for 24.71 % and lipids and lipid-like molecules accounted for 22.86 %.

The differential metabolites were systematically analysed by OPLS-DA. As shown in Figure 3B and C, the OPLS-DA model showed that the R2Y and Q2 values of the differential metabolites of the positive ion model were 0.986 and 0.871, respectively. In the negative ion model, R2Y and Q2 are 0.988 and 0.822, respectively. These data showed that there were highly significant differences in renal tissue metabolite profiles under normal and hypoxia (Figure 3B and C). To ensure the validity of the model, a permutation test was used. Figure 3D and E shows the permutation test of the OPLS-DA model. As the displacement retention decreased both R2 and Q2 of the random model decreased, suggesting that the model does not exhibit an overfitting phenomenon and that it is stable.

Based on univariate analysis, differential metabolites with VIP>1, fold change (FC) >1.5 or FC <0.67, and p-value <0.05 in both positive and negative ion modes were displayed using volcano plots (Figure 4A and B). Specifically, 46 metabolites were upregulated and 45 were downregulated in the positive ion mode, while 25 metabolites were upregulated and 35 were downregulated in the negative ion mode.

Cluster analysis was performed for the metabolites with significant differences. As shown in Figure 4C and D, the cluster heat maps of different metabolites with positive and negative ion pattern properties are shown, respectively. A total of 120 and 79 markedly altered metabolites were identified as differential metabolites (VIP>1, p<0.05) in the positive and negative ion modes, respectively.

KEGG pathway enrichment analyses revealed that differential metabolites were primarily enriched in pathways involved in mTOR signalling (Figure 5A). Differential abundance score is a pathway-based metabolic change analysis method that determines the average change of all metabolites in a certain pathway. Figure 5B depicts the differential abundance score plots for all the enriched metabolic pathways.

Under hypoxic conditions, the expression level of AMP in kidney tissue was downregulated (Figure 6A), whereas the expression level of arginine was upregulated (Figure 6B).

### Combined analysis of differential metabolites and DEGs

A comparison of the pathways involved in the DEGs in the transcriptome and the differentially expressed metabolites in the metabolome revealed 95 metabolic pathways involved in both omics analyses (Figure S7). The mTOR signalling pathway was only enriched based on the KEGG enrichment of differential genes and metabolites analysis (Figure S8). Changes in differentially expressed metabolites and DEGs observed in the mTOR signalling pathway (Figure S9) included downregulated AMP and upregulated arginine under hypoxia.

### Correlation of AMP and arginine with DEGs on the mTOR pathway under chronic hypoxia

**Figure 7:**
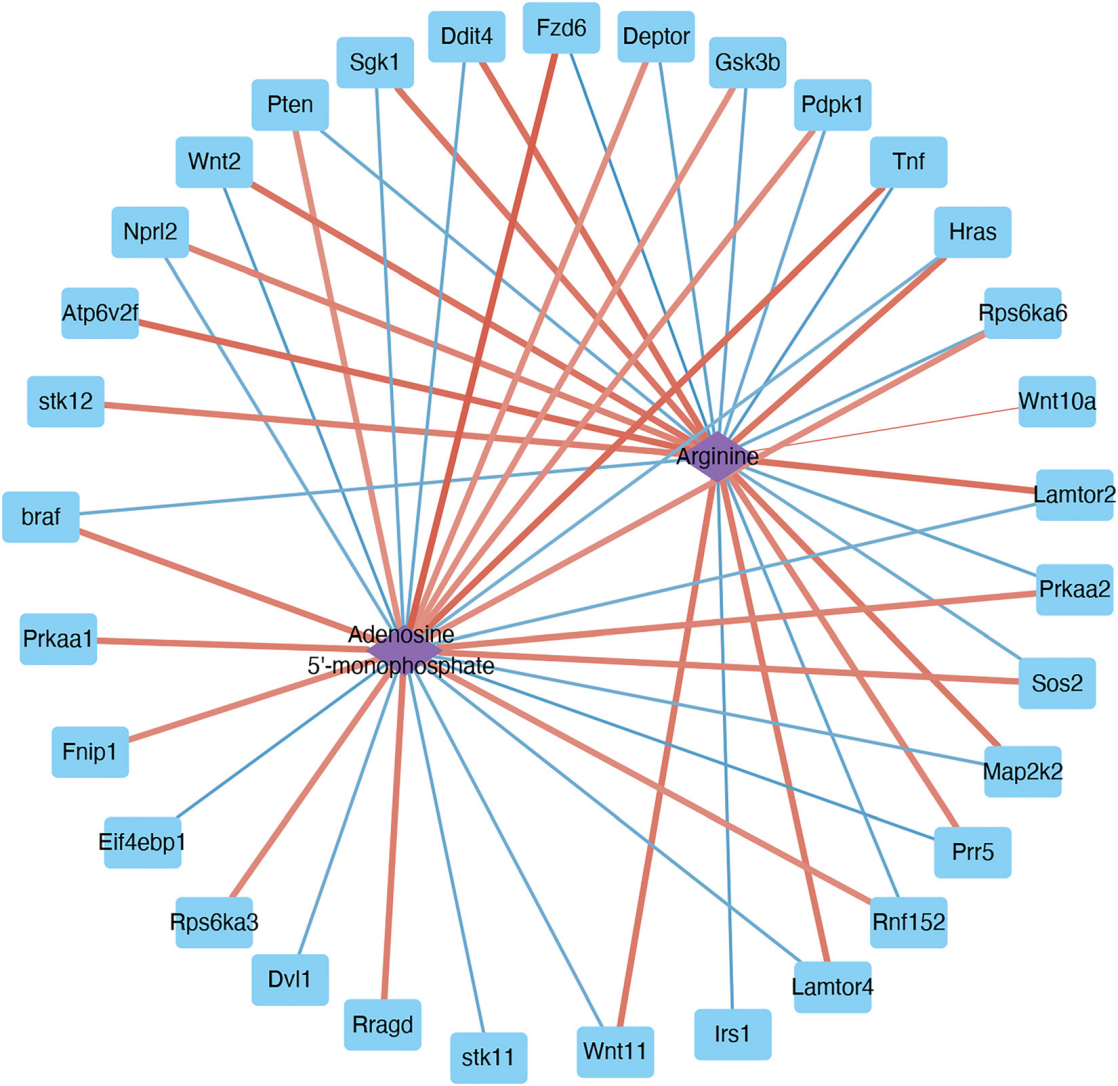
Network analysis of DEGs and differential metabolites. Diamonds represent differential metabolites, rectangles represent differential genes, red lines represent positive correlations, blue lines represent negative correlations, and the thicker the line, the stronger the correlation.

AMP belongs to the subclass of purine ribonucleotides. As shown in Figure 7 and Supplmentary Table 4, AMP was positively correlated with the *Braf, Pten*, *Fzd6, Deptor*, *Gsk3b*, *Pdpk1*, *Tnf*, *Rps6ka6*, *Rragd*, *Prkaa2*, *Rps6ka3*, *Sos2*, *Fnip1*, *Rnf152*, and *Prkaa1*. AMP was negatively correlated with *Stk11, Nprl2, Wnt2, Wnt11, Sgk1, Ddit4, Hras, Wnt10a, Lamtor2, Dvl1, Eif4ebp1, Map2k2, Prr5,* and *Lamtor4*. The VIP, p-value and adjusted P-adj of AMP are presented in Supplmentary Table 5.

As shown in Figure 7 and Supplmentary Table 4, arginine belongs to the subclass of amino acids. Arginine were positively correlated with the *Stk11*, *Atp6v1f, Nprl2*, *Wnt2*, *Wnt11*, *Sgk1*, *Ddit4, Hras*, *Wnt10a*, *Lamtor2,Map2k2*, *Prr5*, and *Lamtor4*. Arginine were negatively associated with *Braf, Pten, Fzd6, Deptor, Gsk3b, Pdpk1, Tnf, Rps6ka6, Prkaa2, Sos2, Rnf152,* and *Irs1*. The VIP, p-value and adjusted P-adj of arginine are presented in Supplmentary Table 5.

## Discussion

In the present study,we found that AMP and arginine levels in renal respond differently to the different altitudes, suggesting an association between hypoxia and metabolic changes in kidney. Through metabolomics and transcriptomic analysis, we found that AMP and arginine within the mTOR pathway were altered under chronic hypoxia.

Homeostasis in an organism requires that cells, tissues, and organs respond appropriately to different environmental signals through tightly controlled signal transduction networks [14]. Oxygen is a key factor in cellular metabolism. Under hypoxia, the organism reprograms metabolic pathways and the corresponding regulatory circuits to ensure cell survival [15], 16]. AMP is a by-product of cellular adenylate kinase reaction [5], and changes in cellular AMP levels are more sensitive maekers of cellular energy status compared to alterations in ADP or ATP levels [17]. In kidney, AMPK is well-documented to play a pivotal role in normal renal physiology and the development of kidney disease, acting as a key regulator that coordinates multiple metabolic pathways to maintain cellular and organismal energy balance. AMPK maintains the energy balance of cells by reducing intracellular ATP demand via inhibiting mTORC1 activity. Our observations revealed that through metabolomic analysis, energy insufficiency in renal tissue under hypoxia was accompanied by decreased AMP content.

mTOR is an evolutionarily conserved sensor of environmental factors with a confirmed association with hypoxia signalling pathways [18], 19]. mTOR exists as two protein complexes, mTORC1 and mTORC2, and plays a regulatory role in cell metabolism, growth, proliferation, survival, and autophagy [20]. Our findings indicated that energy depletion in renal tissue was correlated with the inhibition of the mTORC1 signaling pathway. Arginine is essential for cellular physiology, being involved in numerous cellular metabolic and signaling processes [18], can be transported by solute carriers to activate the downstream mTORC1 signalling pathway [10]. The mTORC1 pathway integrates signals from growth factors, environmental stress, energy status, oxidation, and amino acids to regulate critical physiological processes, including protein and fat synthesis as well as autophagy. Amino acids are indispensable for normal mTORC1 signaling [19], 20]and mediate mTORC1 through the Rag family of proteins [21], 22]. In current study, through combined transcriptomic and metabolomic analyses, we found that renal ATPase expression was upregulated, and we also observed the upregulation of its downstream regulatory complex proteins. Concurrently, hypoxic conditions led to increased renal arginine levels, which was correlated with reduced expression of nitrogen permease regulator-like 3. Collectively, these changes were associated with decreased RagC/D gene expression and subsequent inhibition of the mTORC1 signaling pathway under hypoxia.

Hypoxia has been shown to regulate mTORC1 via DNA damage response-1 (REDD1) [23]. Hypoxia reduced the expression of microrNA-7, which was degraded by binding to the 3-UTR of the REDD1 mRNA. Thus, hypoxia relieved the inhibitory effect of miR-7 on REDD1 expression [24]. mTORC1 signalling under hypoxic conditions is controlled by the ATM-dependent phosphorylation of HIF-1α [25]. In our study, REDD1 activation was observed in renal tissue under hypoxia, and this activation was correlated with inhibition of the mTORC1 signaling pathway; however, a direct regulatory relationship between REDD1 and mTORC1 signaling remains to be further validated.

The most typical substrates of mTORC1 are the ribosomal protein S6 kinase (S6K) and the eukaryotic initiation factor 4E-binding protein 1(4EBP1) [26], 27]. S6K phosphorylates a diverse set of target proteins, many of which promote protein production [28]. Through mTOR-dependent phosphorylation, 4EBP1 dissociates from eIF4E, alleviating the inhibitory effect on eif4E-dependent translation initiation. In our study, hypoxic conditions led to increased expression of S6K and 4E-BP in renal tissue, which may contribute to the promotion of renal protein synthesis. Inhibition of IRS1 by S6K phosphorylation inhibits PI3K activation, forming a negative feedback loop that plays an important role in mTOR regulation [29]. Consistent with this, our results demonstrated that S6K hyperphosphorylation in hypoxic renal tissue was correlated with IRS1 inhibition, which may affect the PI3K/mTORC2 signaling pathway. Independent of PI3k, the Ras/MAPK (mitogen-activated protein kinase) pathway also activates mTORC1 [14]. Some studies reported that the RAS/RAF/MEK pathway regulates the expression of HIF-1α [30]. Our study found that in the renal MAPK pathway under hypoxia, Ras and Mek gene expression was upregulated while Raf gene expression was downregulated through combined transcriptomic and metabolomic analyses. These changes were correlated with alterations in the mTOR pathway and may be involved in the regulation of protein synthesis.

Organisms adapt to the loss of oxygen by regulating the activity of HIF-1 [31]. HIF-1α is downstream of the mTORC1, which is regulated solely by the phosphatidylinositol 3-kinase/protein kinase B (PI3K/Akt) signalling pathway [32]. Furthermore, PTEN gene expression is downregulated in kidney tissue under hypoxia. The loss of PTEN function increases Akt phosphorylation and activity, resulting in decreased apoptosis and increased proliferation signals [33], 34]. Our results indicated that through combined transcriptomic and metabolomic analysis, downregulation of PTEN gene expression in renal tissue was correlated with changes in the mTORC2 signaling pathway via the PI3K/AKT pathway. Serum and glucocorticoid-regulated kinase 1 (SGK1) are involved in cell proliferation and apoptosis [35]. As a downstream gene of mTOR, SGK1 expression was upregulated in hypoxic kidney which may promote cell survival.

### Strengths and limitations

In this study we employed an integrated multi-omics approach combining metabolomics and transcriptomics, which allowed us to systematically explore the metabolic and transcriptional changes of the kidney under chronic hypoxia, and to identify the key roles of AMP and arginine in the mTOR signalling pathway. This multi-dimensional analysis provides a comprehensive perspective on the crosstalk between hypoxia and kidney metabolism, which is more informative than single-omics studies. our findings shed new light on the regulatory mechanism of the mTOR pathway in hypoxic kidney tissue.

Despite these strengths, this study also has several limitations that should be acknowledged. First, the findings are correlational. We observed associations between hypoxia, alterations in metabolites including AMP and arginine, and changes in the mTOR pathway; however, a direct causal relationship among these factors has not been established. Second, functional and phenotypic outcomes related to renal injury were not evaluated in the present study. Third, all experiments were performed using a single-sex animal model and at a single time point. Fourth, direct measurements of mTOR and AMPK phosphorylation were not conducted; therefore, the inhibitory effects of hypoxia on mTORC1 and AMPK activity were inferred from changes in downstream gene expression and metabolite levels, rather than confirmed via phosphorylation assays. Future studies should address these limitations by performing mechanistic experiments to validate causality, assessing comprehensive renal phenotypic outcomes, incorporating both male and female subjects with multiple time-point observations, and conducting direct phosphorylation analyses of mTOR and AMPK to further verify our conclusions.

## Conclusions

In this study, by integrating transcriptomic and metabolomic correlation analyses, we identified enrichment in the mTOR signalling pathway and 33 DEGs; AMP and arginine were identified as differential metabolites. To our knowledge, this is the first study to use multi-omics to investigate the close relationship between hypoxia and renal tissue metabolism. This study provides a basis for future studies on renal metabolism under hypoxic conditions.

## Supplementary Material

Supplementary Material
