# Supplementary material for: Integrating metabolomics and transcriptomics to investigate the effects of changes in renal adenosine 5′-monophosphate and arginine metabolism on the mTOR pathway under chronic hypoxia
Source: Open Med (Wars). 2026 Jun 15;21(1):20261436. doi: 10.1515/med-2026-1436 (PMC13268010; doi:10.1515/med-2026-1436)
Supplement: Supplementary file 1 — Supplementary Material [file j_med-2026-1436_suppl_001.docx]

**Supplementary materials**

1.RNA integrity was verified by RIN assessment, with all samples showing RIN value≥ 6.2, which satisfied the requirements for transcriptome sequencing( STable1).

STable 1 RIN Value of Each Sample

| **Sample name** | **RIN** |
| --- | --- |
| HK-1 | 8.2 |
| HK-2 | 8.6 |
| HK-3 | 7.3 |
| HK-4 | 6.2 |
| HK-5 | 8.8 |
| HK-6 | 7.0 |
| LK-1 | 9.2 |
| LK-2 | 9.2 |
| LK-3 | 9.1 |
| LK-4 | 9.2 |
| LK-5 | 9.4 |
| LK-6 | 9.5 |

2.Raw reads were filtered to obtain high-quality clean reads for subsequent analysis. All samples exhibited excellent sequencing quality, with error rates ≤ 0.05%, Q20 values ≥ 95.08%, Q30 values ≥ 87.30%, and GC content ranging from 44.99% to 48.59%. The data met the quality requirements for reliable transcriptome analysis (STable2).

STable 2 Data quality of each sample

| **Sample** | **Raw_reads** | **Clean_reads** | **Error(%)** | **Q20(%)** | **Q30(%)** | **GC(%)** |
| --- | --- | --- | --- | --- | --- | --- |
| HK-1 | 44521872 | 44513096 | 0.04 | 96.35 | 90.68 | 48.4 |
| HK-2 | 41370682 | 41353878 | 0.04 | 97.09 | 92.27 | 47.38 |
| HK-3 | 45132946 | 45120540 | 0.04 | 96.5 | 91.04 | 48.11 |
| HK-4 | 47528628 | 47521224 | 0.05 | 95.08 | 87.3 | 45.17 |
| HK-5 | 42398054 | 42393208 | 0.04 | 96.34 | 90.59 | 48.59 |
| HK-6 | 43330002 | 43320156 | 0.04 | 96.58 | 91.21 | 46.57 |
| LK-1 | 57186170 | 57177196 | 0.04 | 96.91 | 91.68 | 44.99 |
| LK-2 | 53559730 | 53550148 | 0.04 | 96.93 | 91.72 | 45.09 |
| LK-3 | 58512988 | 58503458 | 0.04 | 96.85 | 91.47 | 45 |
| LK-4 | 60141440 | 60131090 | 0.04 | 97.27 | 92.62 | 45.19 |
| LK-5 | 59400172 | 59390118 | 0.04 | 96.97 | 91.77 | 45.12 |
| LK-6 | 54416308 | 54406690 | 0.04 | 97.14 | 92.22 | 45.02 |

S Table 3 Reads versus reference genome

| **Sample name** | **Total reads** | **Total mapped** | **Multiple mapped** | **Unique mapped** | **Non-splice reads** | **Splice reads** |
| --- | --- | --- | --- | --- | --- | --- |
| HK-1 | 44513096 | 41409699  (93.03%) | 4302827  (9.67%) | 37106872  (83.36%) | 18492111  (41.54%) | 18614761  (41.82%) |
| HK-2 | 41353878 | 38247804  (92.49%) | 5957559  (14.41%) | 32290245  (78.08%) | 16109622  (38.96%) | 16180623  (39.13%) |
| HK-3 | 45120540 | 42149446  (93.42%) | 4439000  (9.84%) | 37710446  (83.58%) | 18796305  (41.66%) | 18914141  (41.92%) |
| HK-4 | 47521224 | 43941178  (92.47%) | 8545364  (17.98%) | 35395814  (74.48%) | 17601501  (37.04%) | 17794313  (37.44%) |
| HK-5 | 42393208 | 39375047  (92.88%) | 4575137  (10.79%) | 34799910  (82.09%) | 17350210  (40.93%) | 17449700  (41.16%) |
| HK-6 | 43320156 | 40411491  (93.29%) | 6626495  (15.30%) | 33784996  (77.99%) | 16813463  (38.81%) | 16971533  (39.18%) |
| LK-1 | 57177196 | 53948877  (94.35%) | 7310645  (12.79%) | 46638232  (81.57%) | 23264507  (40.69%) | 23373725  (40.88%) |
| LK-2 | 53550148 | 50733059  (94.74%) | 6715630  (12.54%) | 44017429  (82.20%) | 21968546  (41.02%) | 22048883  (41.17%) |
| LK-3 | 58503458 | 55294323  (94.51%) | 7422643  (12.69%) | 47871680  (81.83%) | 23876041  (40.81%) | 23995639  (41.02%) |
| LK-4 | 60131090 | 56936698  (94.69%) | 7461303  (12.41%) | 49475395  (82.28%) | 24703804  (41.08%) | 24771591  (41.20%) |
| LK-5 | 59390118 | 56207955  (94.64%) | 7340448  (12.36%) | 48867507  (82.28%) | 24379401  (41.05%) | 24488106  (41.23%) |

3.To assess the quality of the metabolomic profiling, six quality control (QC) procedures were implemented in this study: (1) overlay analysis of total ion chromatograms (TIC) of QC samples(S Figure1-1,1-2); (2) Hotelling-s T2 Range Line Plot(S Figure2-1,2-2); (3) MCC(S Figure3-1,3-2); (4) QC PCA(S Figure4-1,4-2); (5) MultiScatter(S Figure5-1,5-2); (6) QCRSD_curve(S Figure6-1,6-2).


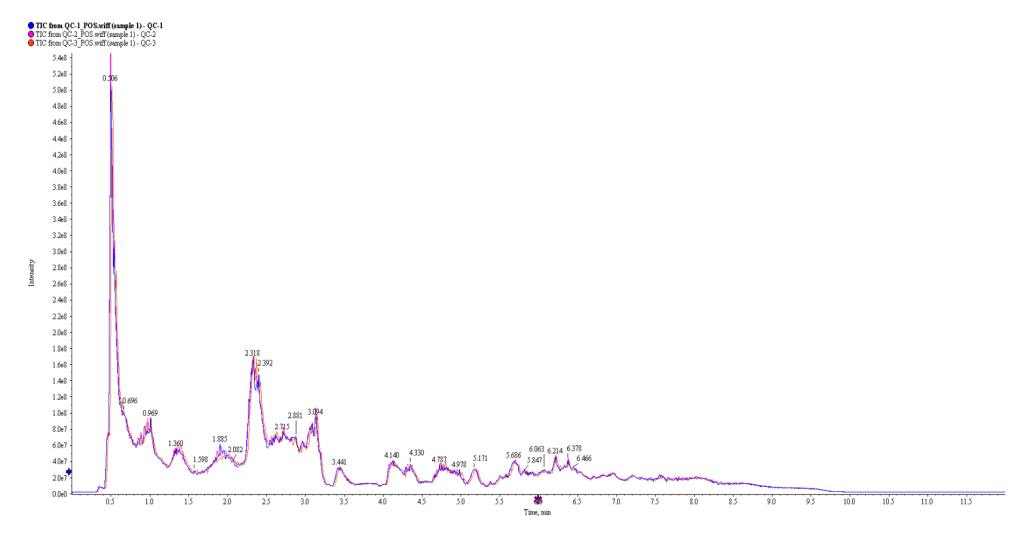


**SFigure1-1** Total ion chromatograms overlapping spectrum of QC samples in positive ion mode


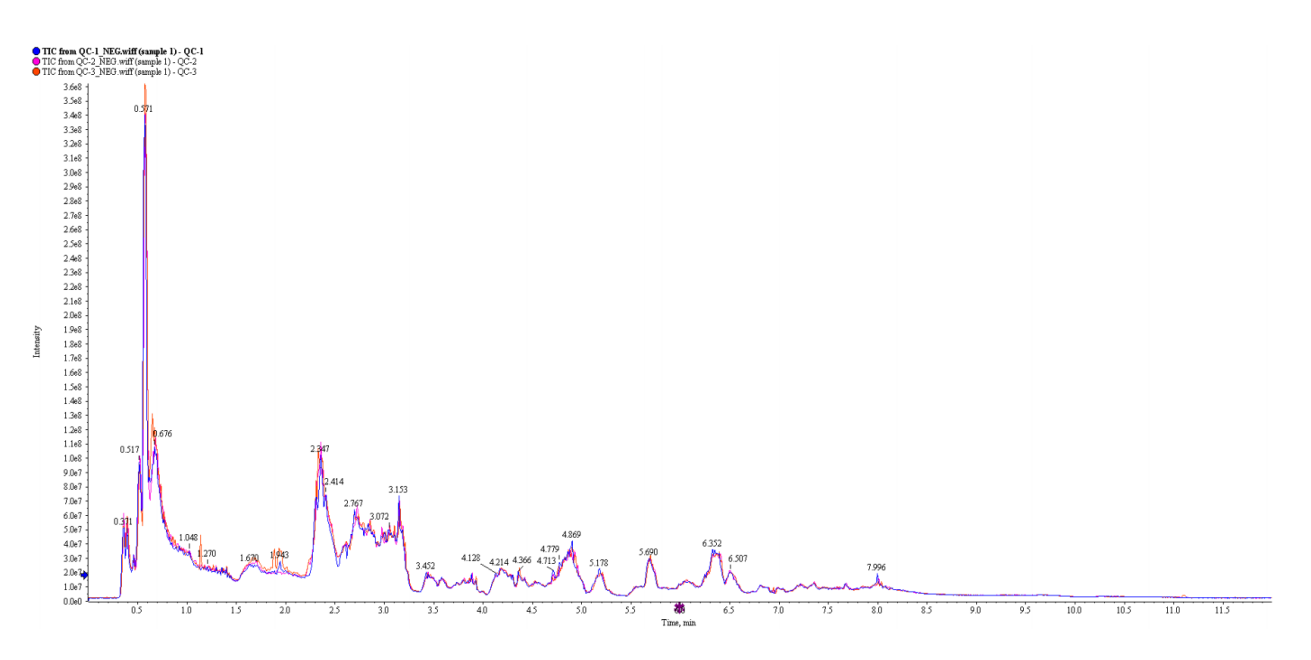


**SFigure1-2** Total ion chromatograms overlapping spectrum of QC samples in negative ion mode


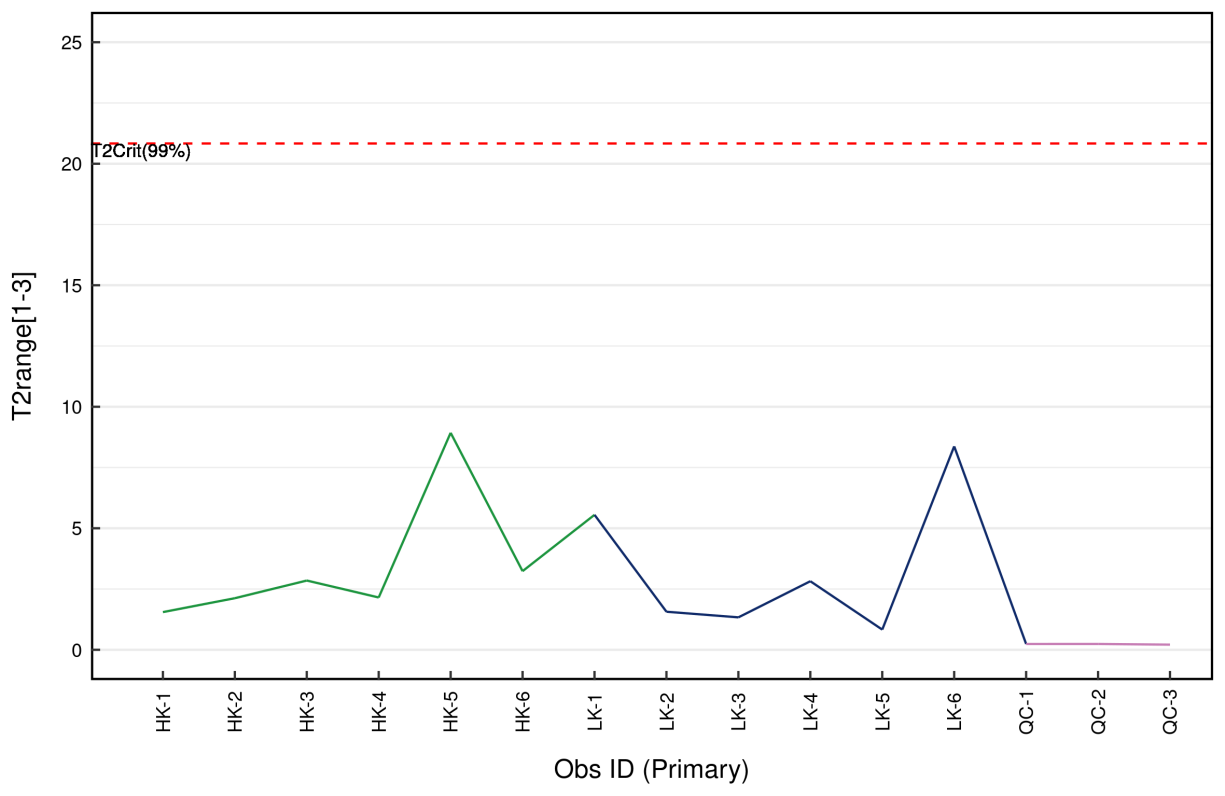


**SFigure2-1** Hotelling-s T2 Range Line Plot in negative ion mode

**
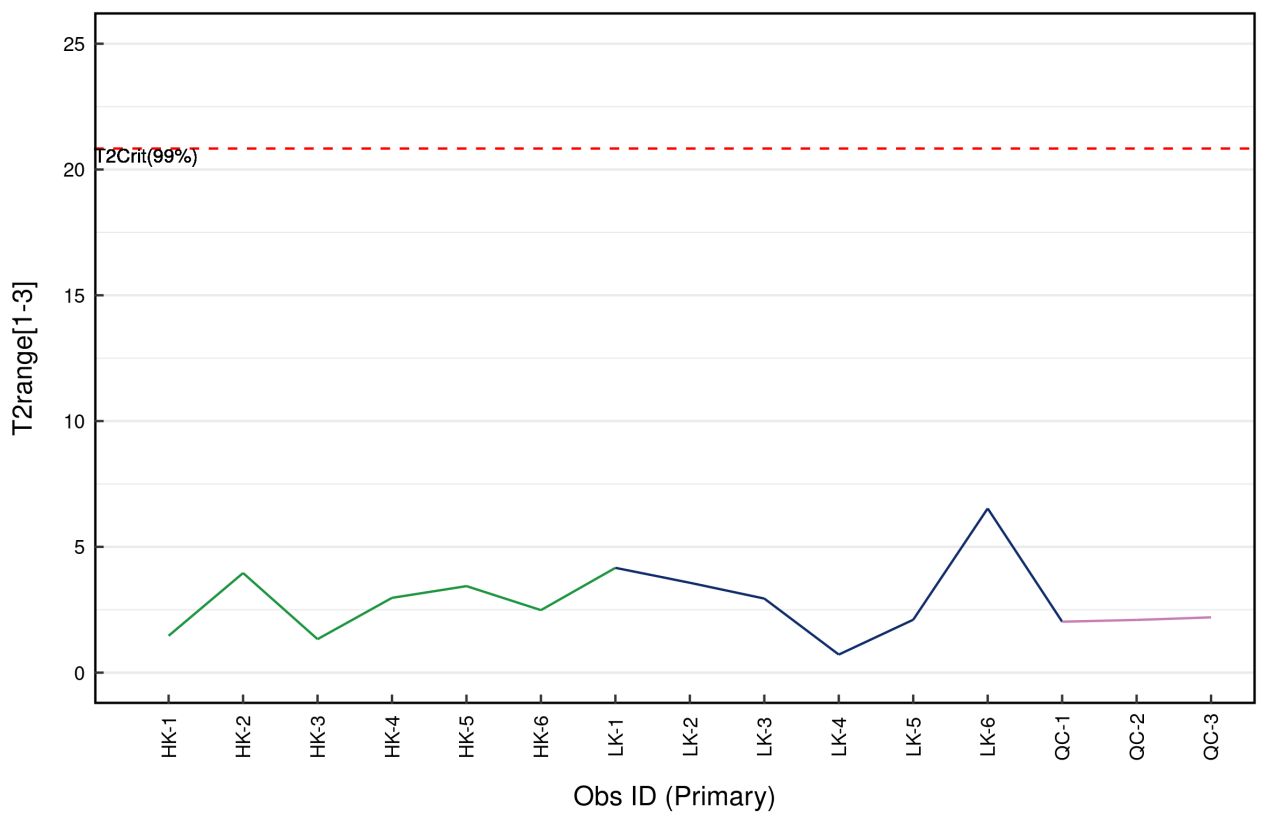
**

**SFigure2-2** Hotelling-s T2 Range Line Plot in positive ion mode


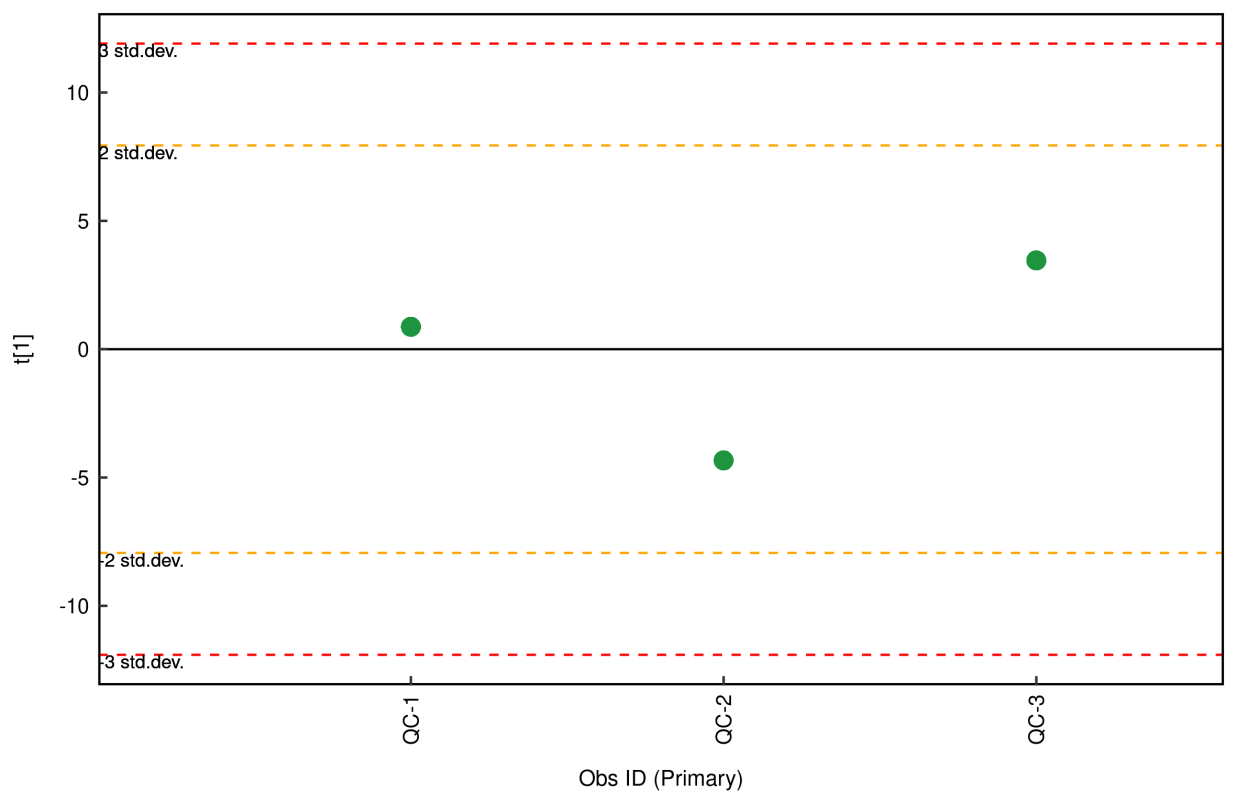


**SFigure3-1** MCC in negative ion mode


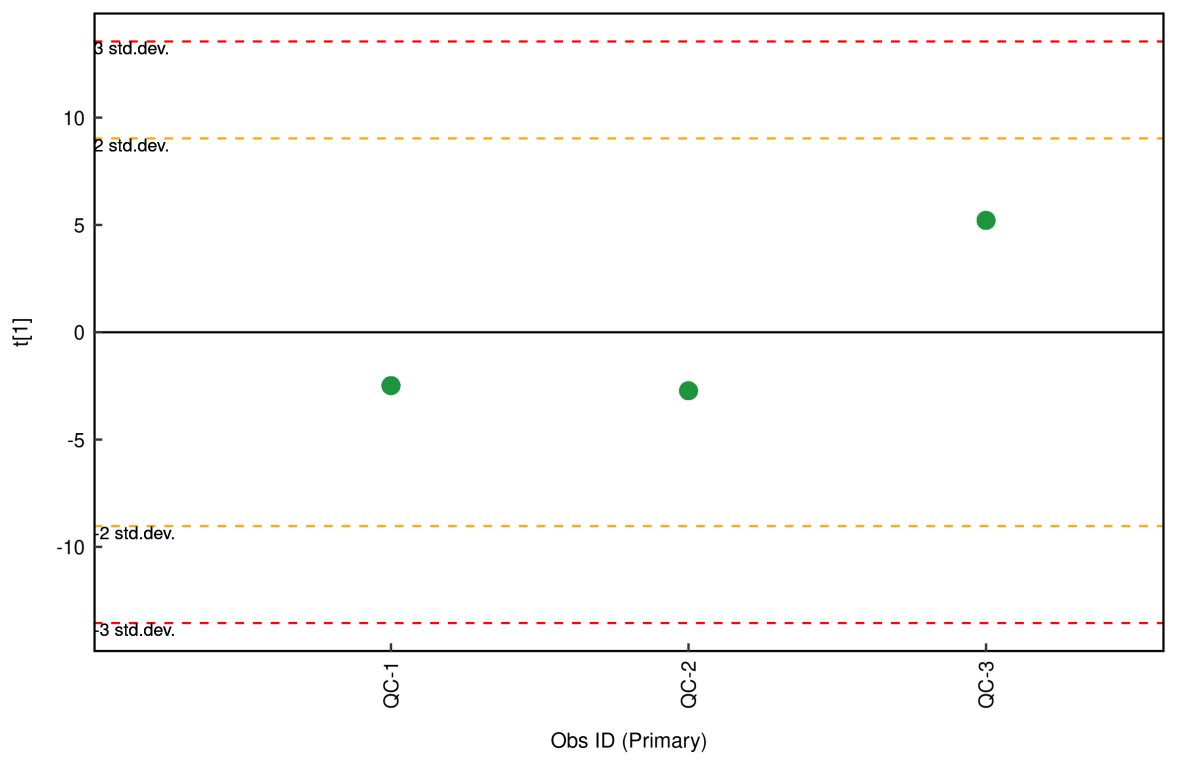


**SFigure3-2** MCC in positive ion mode

**
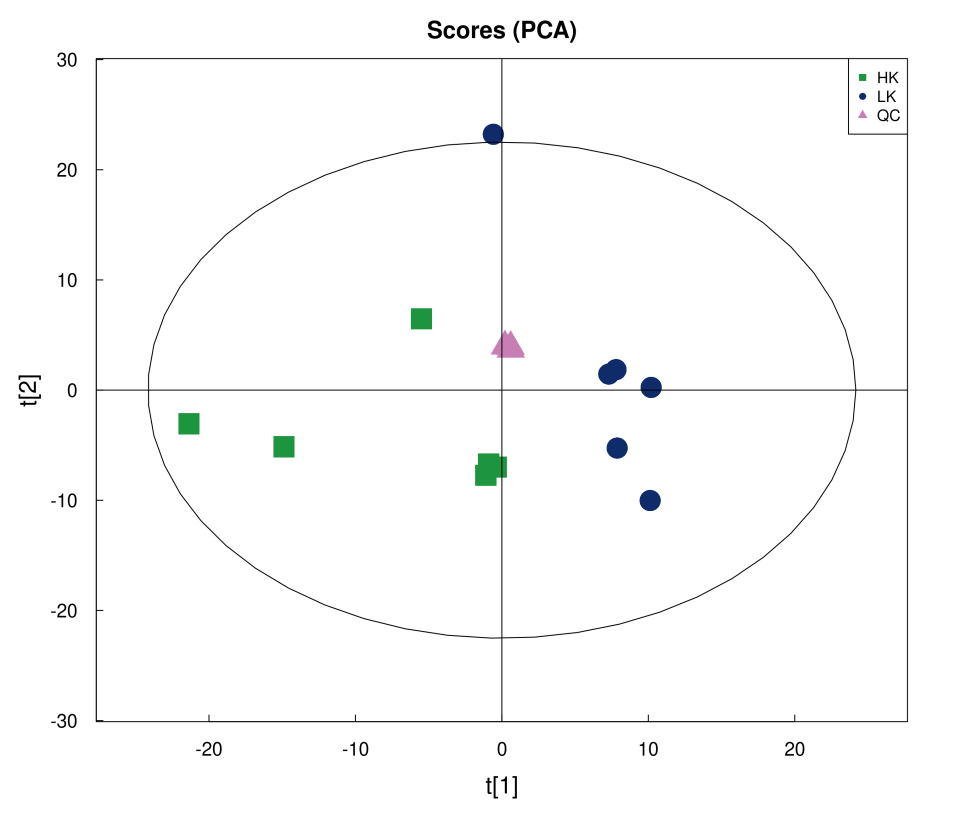
**

**SFigure4-1** QC PCA negative ion mode


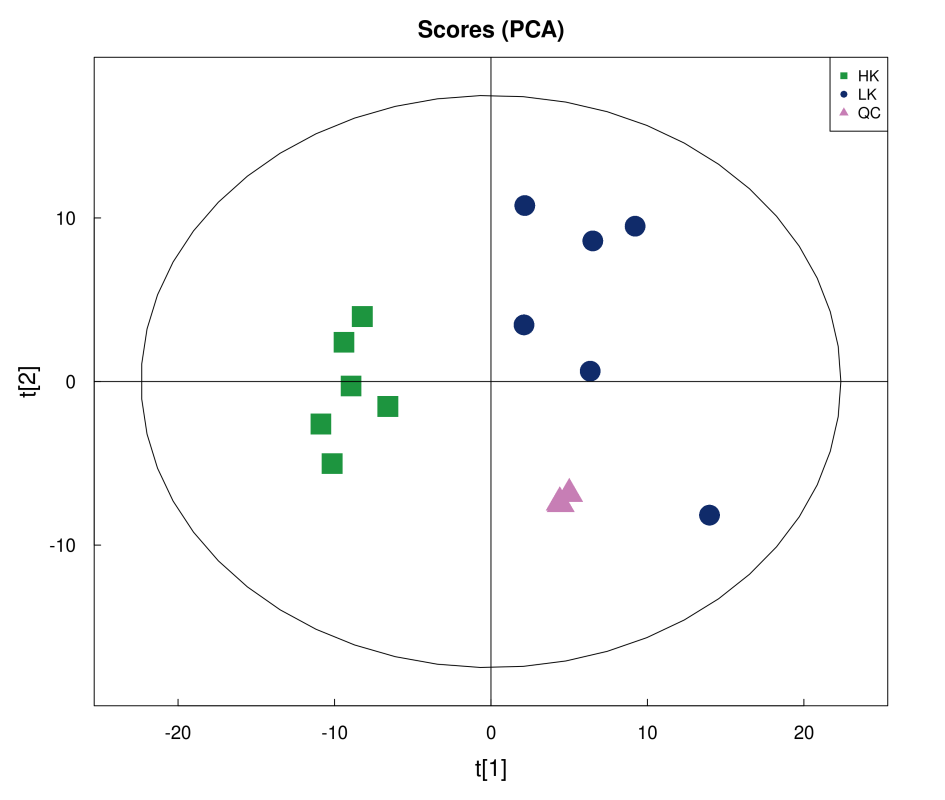


**SFigure4-2** QC PCA in positive ion mode


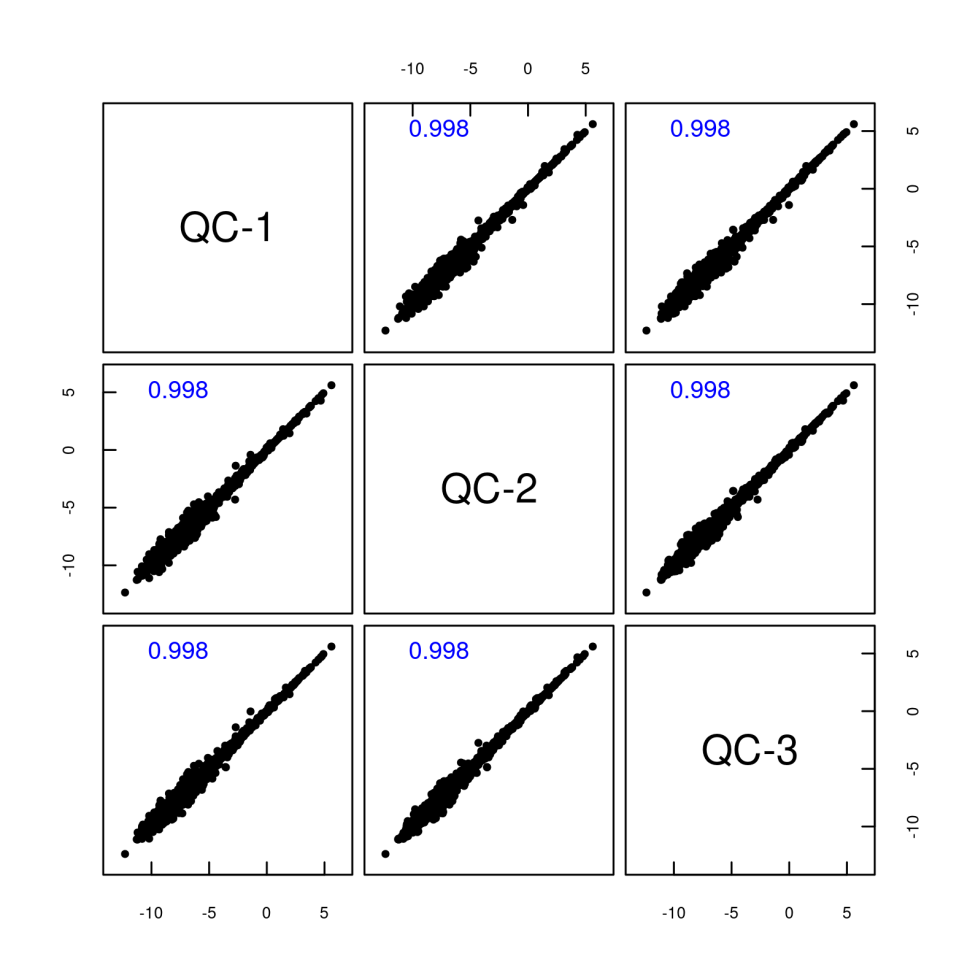


**SFigure5-1** MultiScatter in negative ion mode


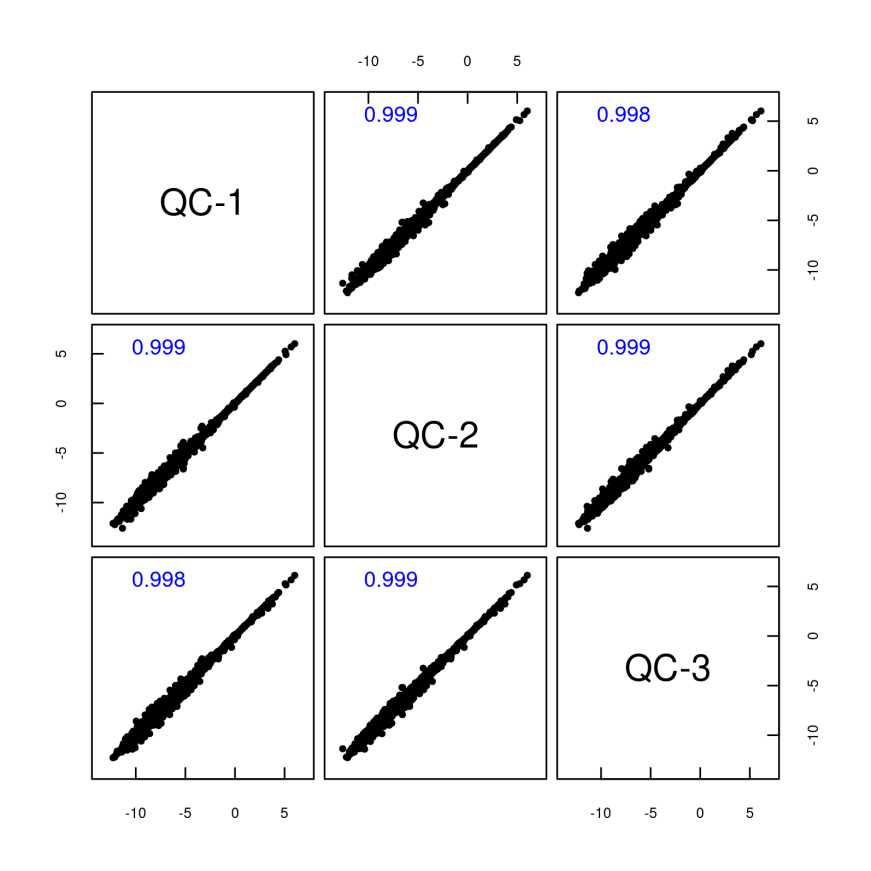


**SFigure5-2** MultiScatter in positive ion mode


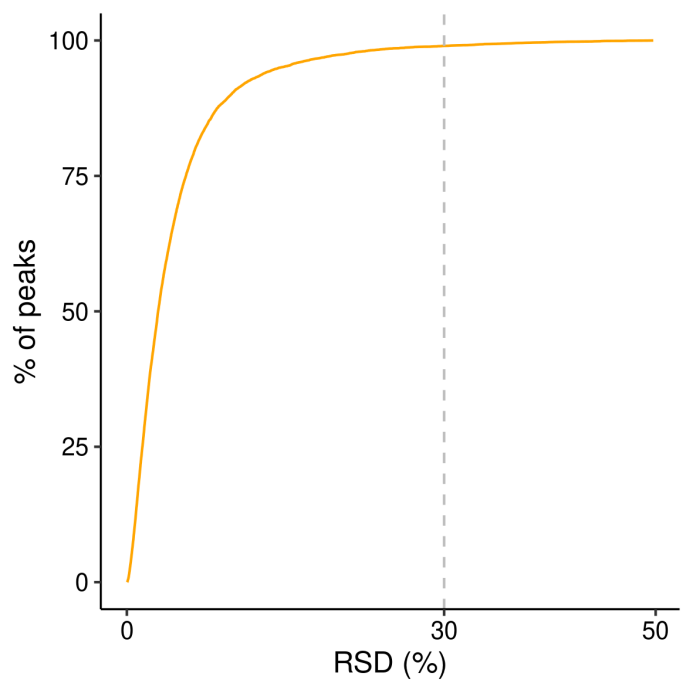


**SFigure6-1** QCRSD_curve in negative ion mode


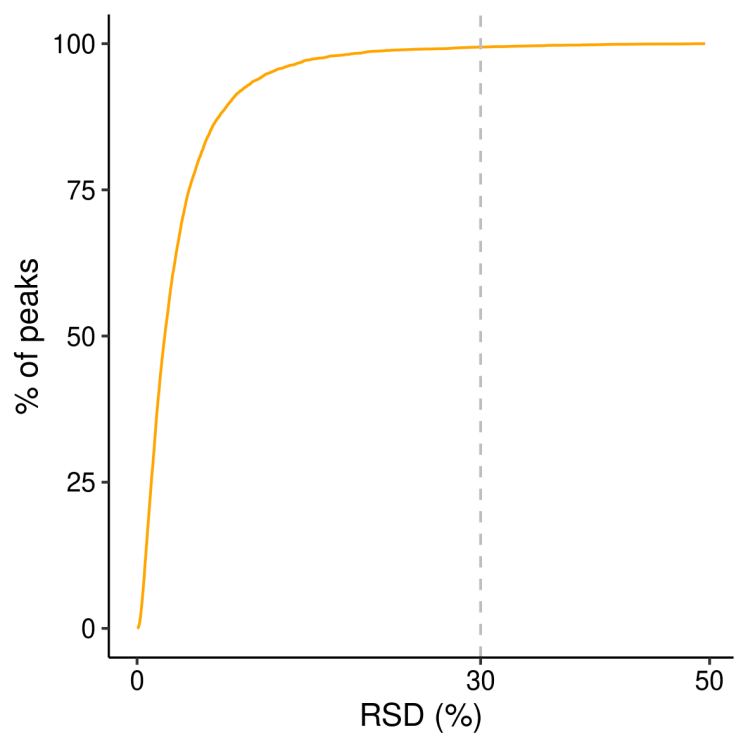


**SFigure6-2** QCRSD_curve in positive ion mode

**4.List of correlations between 2 differential metabolites and 33 differentially expressed genes(STable4)**

**STable4** Correlation between 33 DEGs and AMP or arginine

5.List of OPLS-DA VIP, *P*-value and *P*-adj for two differential metabolites(STable5)

**S Table5** VIP and P-values of AMP and Arginine metabolites

| **metabolites** | **VIP** | ***P*-value** | ***P*-adj** |
| --- | --- | --- | --- |
| AMP | 1.108 | 0.008 | 0.021 |
| Arginine | 3.029 | 0.006 | 0.016 |

| **Gene** | **Adenosine 5'-monophosphate** | | | **Arginine** | | |
| --- | --- | --- | --- | --- | --- | --- |
|  | **Coefficient** | ***P* value** | **Lable** | **Coefficient** | ***P* value** | **Lable** |
| *Braf* | 0.727 | 0.035 | pos | -0.672 | 0.045 | neg |
| *Stk11* | -0.709 | 0.037 | neg | 0.733 | 0.039 | pos |
| *Atp6v1f* | -0.609 | 0.055 | - | 0.842 | 0.025 | pos |
| *Nprl2* | -0.655 | 0.041 | neg | 0.709 | 0.040 | pos |
| *Wnt2* | -0.809 | 0.026 | neg | 0.806 | 0.027 | pos |
| *Pten* | 0.627 | 0.048 | pos | -0.685 | 0.041 | neg |
| *Wnt11* | -0.673 | 0.040 | neg | 0.770 | 0.034 | pos |
| *Sgk1* | -0.700 | 0.039 | neg | 0.782 | 0.034 | pos |
| *Ddit4* | -0.709 | 0.037 | neg | 0.818 | 0.025 | pos |
| *Fzd6* | 0.918 | 0.004 | pos | -0.891 | 0.012 | neg |
| *Deptor* | 0.636 | 0.047 | pos | -0.745 | 0.037 | neg |
| *Gsk3b* | 0.627 | 0.048 | pos | -0.721 | 0.040 | neg |
| *Pdpk1* | 0.655 | 0.041 | pos | -0.685 | 0.041 | neg |
| *Tnf* | 0.855 | 0.013 | pos | -0.806 | 0.027 | neg |
| *Rps6kb2* | -0.618 | 0.051 | - | 0.600 | 0.070 | - |
| *Hras* | -0.636 | 0.047 | neg | 0.794 | 0.031 | pos |
| *Rps6ka6* | 0.645 | 0.045 | pos | -0.685 | 0.041 | neg |
| *Wnt10a* | -0.564 | 0.073 | neg | 0.927 | 0.004 | pos |
| *Lamtor2* | -0.655 | 0.041 | neg | 0.818 | 0.025 | pos |
| *Rragd* | 0.736 | 0.034 | pos | -0.624 | 0.059 | - |
| *Prkaa2* | 0.709 | 0.037 | pos | -0.709 | 0.040 | neg |
| *Dvl1* | -0.682 | 0.040 | neg | 0.612 | 0.065 | - |
| *Rps6ka3* | 0.673 | 0.040 | pos | -0.624 | 0.059 | - |
| *Eif4ebp1* | -0.791 | 0.025 | neg | 0.636 | 0.055 | - |
| *Wnt6* | -0.451 | 0.164 | - | 0.608 | 0.066 | - |
| *Sos2* | 0.718 | 0.037 | pos | -0.661 | 0.048 | neg |
| *Map2k2* | -0.673 | 0.040 | neg | 0.770 | 0.033 | pos |
| *Fnip1* | 0.673 | 0.040 | pos | -0.648 | 0.051 | - |
| *Prr5* | -0.800 | 0.025 | neg | 0.697 | 0.041 | pos |
| *Rnf152* | 0.664 | 0.041 | pos | -0.721 | 0.040 | neg |
| *Lamtor4* | -0.691 | 0.040 | neg | 0.758 | 0.035 | pos |
| *Prkaa1* | 0.736 | 0.034 | pos | -0.503 | 0.140 | - |
| *Irs1* | 0.600 | 0.058 | - | -0.770 | 0.034 | neg |

6.Integrated transcriptomic and metabolomic analysis revealed that 95 overlapping molecules were commonly regulated at both transcript and metabolite levels. Specifically, 236 molecules were uniquely altered in the transcriptome, while 4 molecules were specifically changed in the metabolome(S Figure7).


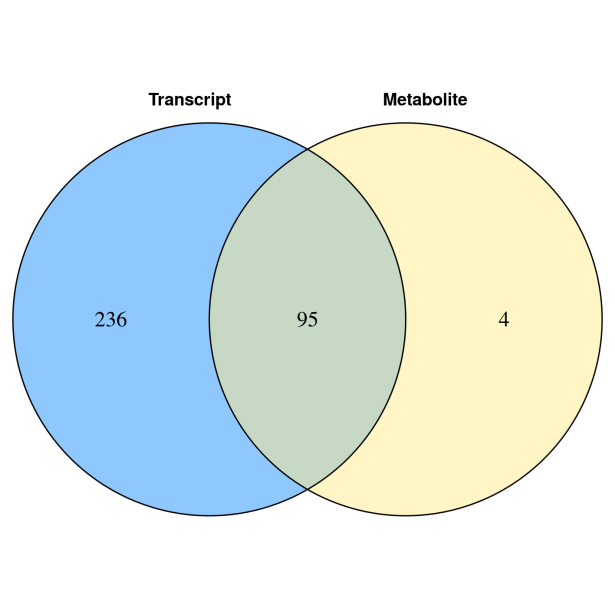


**SFigure7. Venn diagram of differential genes and differential metabolites involved in pathways**：

7.The bar plot shows the enrichment significance of the mTOR signaling pathway.The mTOR signaling pathway was significantly enriched in the metabolome, while it also showed a notable enrichment trend in the transcriptome(S Figure8).


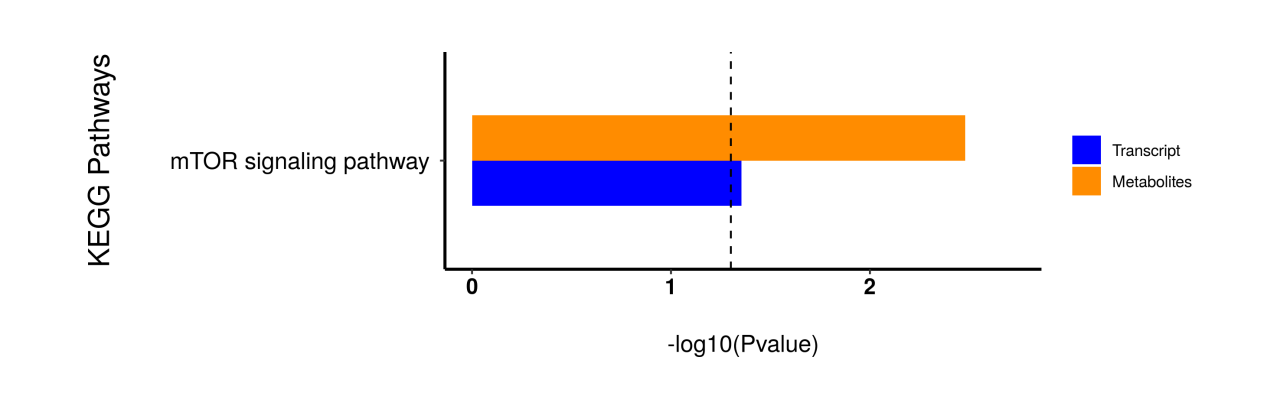


**S Figure8.Integrated KEGG pathway enrichment analysis of transcriptomic and metabolomic**

8.This KEGG pathway map illustrates the molecular alterations in the mTOR signaling pathway identified by combined transcriptomic and metabolomic profiling(S Figure9).


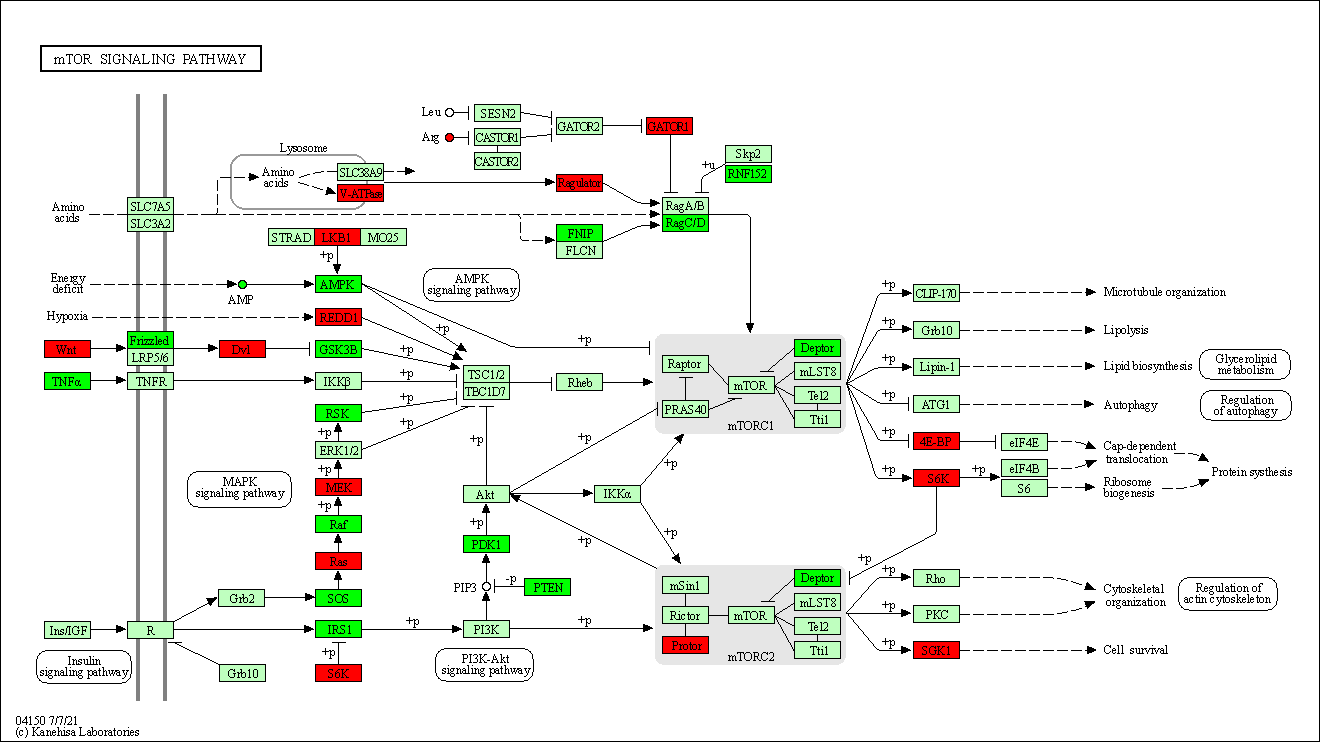


**S Figure9. KEGG pathway map of the mTOR signaling pathway.**
